# Acute effect of resistive aquatic high-intensity interval training on metabolic costs in adults

**DOI:** 10.3389/fspor.2024.1421281

**Published:** 2024-10-14

**Authors:** Manny M. Y. Kwok, Shamay S. M. Ng, Y. M. Ng, Gordon C. C. Tan, P. P. Huang, Y. Zhang, Billy C. L. So

**Affiliations:** ^1^ Gait and Motion Analysis Laboratory, Department of Rehabilitation Sciences, The Hong Kong Polytechnic University, Hong Kong, Hong Kong SAR, China; ^2^ Rehabilitation Division, The Hong Kong Society for Rehabilitation, Hong Kong, Hong Kong SAR, China; ^3^ Rehabilitation Sciences Department, Jiangbin Hospital of Guangxi Zhuang Autonomous Region, Guangxi, China

**Keywords:** aquatic exercise, high interval, resistive exercise, physical activity, metabolism

## Abstract

**Background:**

The effects of Aquatic High-Intensity Interval Training (AHIIT) and resistive AHIIT (Resistive AHIIT) to improve metabolic responses were not yet known.

**Objective:**

This study was to compare the metabolic responses and perceived effort in young healthy adults in a single session of AHIIT and resistive AHIIT.

**Methods:**

20 healthy subjects (9 females, 11 males) performed a stationary running at a matched exercise intensity prior AHIIT and resistive AHIIT [10 × 1-min bouts of stationary running at 90% maximum heart rate (HR max) separated by 1-min active recovery] to examine the metabolic and cardiometabolic outcomes. Mixed effects models were applied to analyze the effects of group, time, and the interaction between group and time on both outcomes. The level of correlations between metabolic variables was checked by Pearson's linear correlation.

**Results:**

There are significant differences on pre and post resting energy expenditure (REE) within both AHIIT and resistive AHIIT groups (*p* < 0.01) respectively as well as the subjective rate of perceived exertion (RPE) (*p* < 0.01) within RAHIIT group. A moderate correlation found on respiratory exertional ratio (RER) and RPE in resistive AHIIT (r = 0.534). No significant differences between groups in terms of HR max, mean heart rate (HR mean), peak oxygen consumption (VO_2_ peak) and total energy expenditure (TEE) (*p* = 0.50, *p* = 0.48, *p* = 0.81, *p* = 0.59).

**Conclusion:**

Resistive AHIIT provides comparable benefits of metabolic outcomes with AHIIT. Comparable results allowed AHIIT and resistive AHIIT prescriptions precisely.

## Introduction

The sedentary lifestyle has been proven to be associated with weight changes and obesity, increasing the risk of deaths caused by comorbidities such as Type 2 diabetes mellitus, dyslipidemia, hypertension, and obstructive sleep apnea (1). Adults often face barriers to participating in exercise, such as tiredness, physical discomfort, stress, and time constraints (2). To address these challenges, high-intensity interval training (HIIT) is promoted as a way to enhance basal metabolism, exercise enjoyment, and time efficiency (3).

HIIT involves alternating between high-intensity exercise and rest or moderate-intensity active recovery periods. The exercise intensity during HIIT reaches 85%–90% of maximal heart rate (HR) or over 90% of maximal oxygen consumption (VO_2_) during the work phase (4). HIIT can also be performed in a water-based environment, and the properties of water may lead to more favorable training effects compared to land HIIT (LHIIT) (5).

Water possesses several properties that contribute to its training benefits, including buoyancy, hydrostatic pressure, drag force, and thermodynamics (6). Buoyancy effectively reduces a person's body weight when immersed in water, with 40% of weight offloaded when the umbilicus is immersed and 60% when the xiphoid is immersed (7, 8). Hydrostatic pressure displaces blood from the venous and lymphatic system back to the heart, increasing stroke volume (9). Drag force refers to the resistance experienced by an object in water due to its shape and size, and it is directly proportional to the amount of resistance encountered (10). Water is also an efficient heat conductor, transferring heat 25 times faster than air (11). It can easily deliver temperature to immersed body parts, with typical hydrotherapy pools operating within the range of 33.5–35.5 degrees Celsius (12).

Evidence suggests that aquatic HIIT (AHIIT) elicits more desirable training effects than LHIIT. Studies have shown that, at the same exercise intensity, AHIIT is associated with lower perceived exertion during and after training compared to LHIIT, as measured by the Borg Rating of Perceived Exertion Scale (13). AHIIT has also demonstrated faster recovery than LHIIT, as indicated by a more than 10% reduction in post-exercise heart rate reserve (14). Moreover, AHIIT has favorable effects on oxygen consumption and energy expenditure (15). In a study conducted by Kwok et al. (16), AHIIT was found to result in a decrease in maximal heart rate (HR max) and work and recovery heart rates compared to LHIIT (17). These findings suggest that a water-based environment offers more favorable training outcomes for HIIT.

However, to the best of our knowledge, no current study has investigated the variation of AHIIT and compared which variation yields the best training effects on basal metabolism. Basal metabolism refers to the energy expenditure of a person at rest and reflects their health status and physical fitness to some extent (18). Previous research has shown that high-intensity interval resistance training increases post-exercise resting energy expenditure (REE) and respiratory ratio, indicating an improvement in basal metabolism and fat oxidation (19). Aquatic resistive training has been widely examined in recent year, which resulted in increased in maximal dynamic strength of the lower limbs (20). Furthermore, resistive exercises increase power and, as a consequence, improve physical fitness, health, and functional autonomy (21). The density of water generates increased muscle strength because water generates resistance 900 times greater than in air (22). An aquatic resistance device may be deployed to ride along the resistance caused by the drag force when moved in water. The use of resistive devices generates muscular tension when moved in opposition to the water. Hence, the drag force is responsible for the resulting resistance during the use of the resistive device and can be defined as a resistant force opposite to the direction of movement of an object, which can occur both in front of and behind the object that is moved (23). The magnitude of the drag force depends mainly on the surface area and the shape of the device but is also determined by the velocity of movement (cadence) such that an increase in the velocity of movement exponentially increases the drag force. Drag force imposed by water enables a greater recruitment of motor units with higher excitation thresholds activated. The effects of resistance training on resting metabolic rate (RMR) are less clear and has the potential to increase RMR and daily energy expenditure (24). This suggests that performing resistive AHIIT may potentially have a greater impact on resting metabolic rate than AHIIT. In the context of aquatic exercise, resistance can be applied by increasing cadence and limb surface area. In this study, resistive AHIIT is performed by wearing resistance boots to increase the surface area of the lower limbs.

Hence, AHIIT has demonstrated superior improvements in cardio-metabolism compared to LHIIT, and resistive AHIIT represents a variation of AHIIT that potentially yields greater training effects on basal metabolism. However, no studies have been conducted to examine the training effects of resistive AHIIT on basal metabolism or to compare the metabolic effects of AHIIT and resistive AHIIT. Therefore, the research question for this study is twofold: (1) What is the exercise effect of resistive AHIIT on basal metabolism? and (2) What are the differences in basal metabolism between AHIIT and resistive AHIIT in healthy adults? The objective of this study is to investigate metabolic responses after a single bout of resistive AHIIT and to compare the differences between AHIIT and resistive AHIIT. It is hypothesized that resistive AHIIT will result in comparable or superior basal metabolism compared to AHIIT.

## Materials & methods

### Study design and procedure

This study is a randomized crossover study aimed at comparing the cardiorespiratory and metabolic responses of young, healthy adults performing AHIIT and resistive AHIIT, with a focus on matching intensities between aerobic and interval resistance training.

Prior to participating in the study, subjects underwent a screening interview and a familiarization period. Informed consent was obtained from all subjects. The screening process involved a standard health questionnaire (International Physical Activity Questionnaire), as well as measurements of resting heart rate, blood pressure, body mass, and height, in order to assess the subjects’ habitual physical activity levels (25). All subjects completed a familiarization session before the study, during which the details of the exercises, including the range of movements and the use of a mouthpiece and Hans Rudolph valve, were explained. A registered aquatic physiotherapist provided feedback and instruction to the subjects during the trials and sessions.

**Figure 1:**
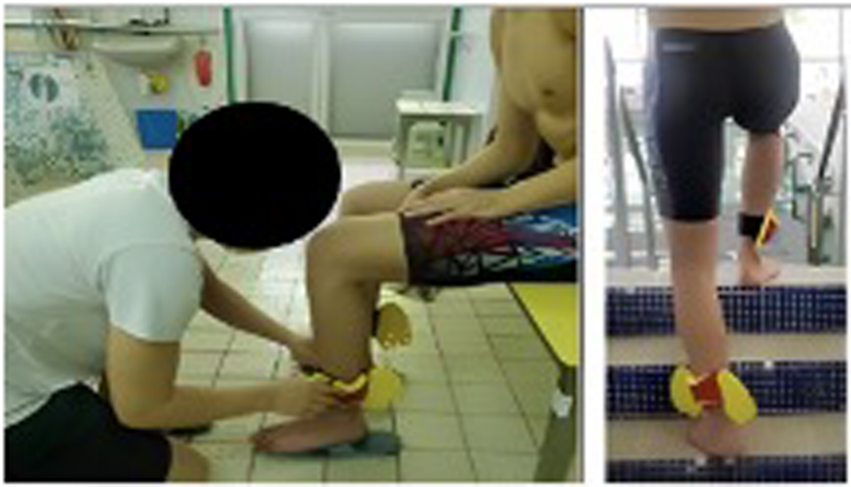
Details of resistance boots and standardization in wearing the boots.

The HIIT exercise, specifically stationary running, was performed at an intensity set at 90% of the subjects’ maximum heart rate (HRmax), as determined by an incremental test. This intensity was used to establish individualized cadence for later high-intensity aerobic interval training. Both AHIIT and resistive AHIIT exercises were performed at the same matched intensity, measured by cadence (130–150 beats per minute), set by a digital metronome (MA-30, KORG; Tokyo, Japan). The exercises took place in a heated pool with a water temperature of 34°C and a depth of 0.95–1.40 m at a hydrotherapy Pool. Each exercise session lasted for 10 min and was preceded by a 5-minute warm-up and followed by a 5-minute cool-down. During the 10-minute exercise period, there were 5 sets of stationary running, with each set consisting of 1 min at 90% HRmax followed by 1 min of dynamic rest at 70% HRmax. The AHIIT and resistive AHIIT protocols were identical in setup, except for the use of resistance boots in the resistive AHIIT protocol (Figure 1) and the cadence for the resistive AHIIT exercises.

**Figure 2:**
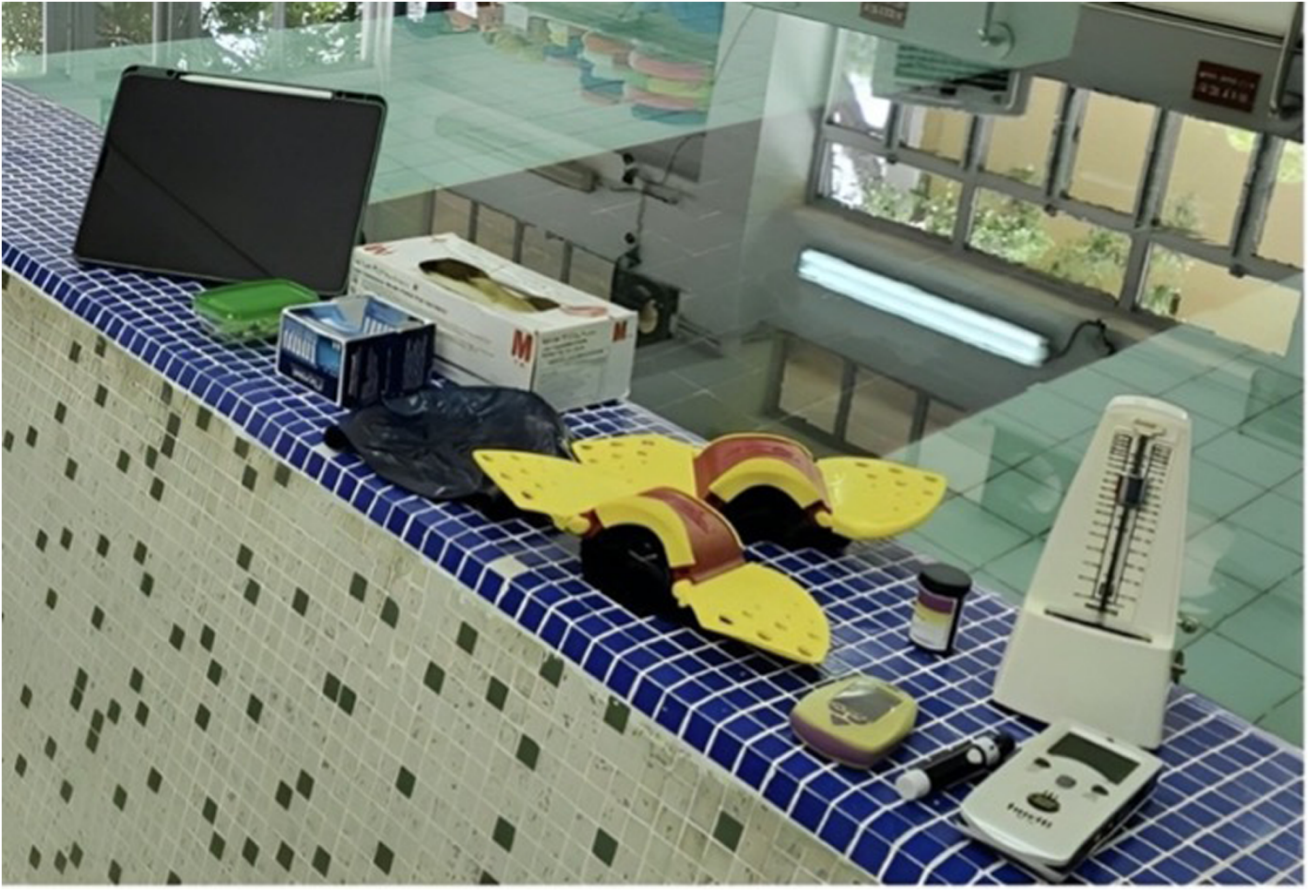
Set up of AHIIT and Resistive AHIIT.

**Figure 3:**
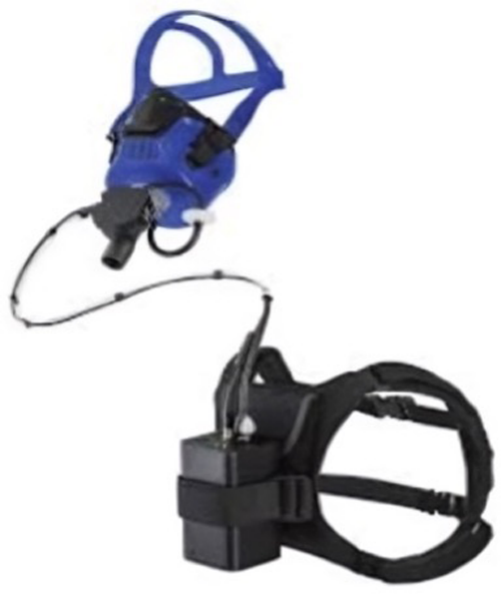
PNOE and Polar OH1.

To measure the subjects’ resting energy expenditure (REE) and respiratory exchange ratio (RER), a portable metabolic analyzer (PNOE Cardio-metabolic Analyzer, Yale Street, USA) was used (Figure 2). The analyzer collected breath-by-breath data by means of an aqua trainer adapter covering the flow sensor, which measured inhaled and exhaled air. The RER and REE (breath-by-breath oxygen consumption) were calculated using the device. The gas analysis system was calibrated before each test using standard reference gases and a 3-L syringe (Model 5530, Hans Rudolf, Kansas City, MO). During each session, subjects wore a breathing mask for the collection of expiratory gas. The set included a facemask covering the nose and mouth, a cap, and a flow sensor, which directed the exhaled air to the PNOE device. Heart rate was continuously monitored and recorded at a frequency of 1 Hz using a heart rate sensor (Polar OH1, Kempele, Finland), (Figure 3). Perceived exertion was assessed during the intervention using the Borg 15-point rate of perceived exertion (RPE) scale (20). Subjects were shown an A3-format plastic panel (297mm × 420 mm) representing the Borg's scale for this purpose.

### Sampling and sample size calculation

Subjects were recruited through convenience sampling at the Hong Kong Polytechnic University. The sample size was determined based on the primary outcome of a previous study that compared REE and RER in aquatic aerobic and resistive training (26). Effect sizes (ES) were calculated by Cohen's d. Using the G*power software and based on the effect size 0.28 obtained, the primary outcome (REE) assuming a 5% type I error and 80% power, the sample size of 17 or more subjects per group was calculated for the primary outcome, assuming a 5% type I error and 80% power. Considering an estimated attrition rate of 20%, a total enrolled sample size of 20 was determined to ensure sufficient statistical power.

### Ethics approval and informed consent

Prior to data collection, both the ethical approval and informed consent sheets were obtained.

### Outcome measures

The primary outcomes of this study were REE and RER. REE accounts for 60%–75% of daily energy expenditure, and was estimated by the gas analyzer PNOE. It measured resting energy expenditure via indirect calorimetry. It measured the amount of oxygen inhaled (VO_2_) and the amount of carbon dioxide exhaled (VCO_2_) (19). RER is the ratio of carbon dioxide production to oxygen uptake, directly measured using VCO_2_ and VO_2_ helps determine the proportion of carbohydrates and fats used for energy consumption at rest (27). Values of 0.7, 0.8, and 1.0 represent respiratory quotient values for fat, protein, and carbohydrates, respectively (27, 28).

The secondary outcomes included VO_2_ peak, HR max, HR mean, TEE, and RPE. For male subjects, a good level of VO_2_ peak is typically in the range of 42–46 ml/kg/min, while for female subjects, it falls within 33–37 ml/kg/min (29). VO_2_peak, HR max, HR mean, and TEE were selected to reflect the cardiometabolic aspects of the subjects, while RPE was monitored to observe their perceived exertion.

### Inclusion and exclusion criteria

Subjects were recruited through convenience sampling. The inclusion criteria were as follows: (1) age between 18 and 35, (2) clinically healthy, (3) no musculoskeletal, bone and joint, cardiac, or pulmonary distress requiring medication, and (4) not pregnant. The exclusion criteria were: (1) presence of cardiovascular, respiratory, musculoskeletal, orthopedic, or metabolic problems, neurological pathology, recent lower limb fracture or surgical intervention within the past six months, (2) hydrophobia, and (3) other pathologies that would hinder participation in aquatic exercise. A total of 20 healthy subjects (9 females, 11 males) were recruited, and all 20 subjects (9 females, 11 males) completed the study.

### Data collection procedure

The data collection procedure involved subjects participating in an incremental exercise test (stationary run) while immersed in the test pool. The incremental test was performed prior to the exercise interventions to confirm an individualized cadence required at a matched level of exercise intensity (stationary running at 90% with 1-min active recovery at 70% HR max in between) in each condition. Instructions for stationary running directed participants to flex the hip and knee to as close to 90° as comfortable and control allowed and then push to straighten up hip and knee. Prior to testing, all exercises were demonstrated first, then practiced once. Participants was monitored continuously and recorded at a frequency of 1 Hz by a HR sensor (Polar OH1, Kempele, Finland). The HR sensor used has been shown to provide valid and reliable HR data (12). During the incremental test, gas exchange data were obtained by a portable metabolic device PNOE. The PNOE device was operated by a breath-by-breath mode which continuously measures volume and determines expired gas concentrations simultaneously. It was calibrated prior to each session according to manufacturer's specifications. PNOE has been validated in previous research, as compared to a validated stationary metabolic cart (COSMED QUARK-CPET) (30). The incremental protocol increased the exercise load from 85 beats per minute (bpm) and increased the cadence by 15 bpm every 2 min for each progression (31). A metronome (Intelli IMT 300, Japan) was used to monitor the speed of movements throughout the trial. The HR, VO_2_, RPE per minute were recorded. VO_2_ max was considered to be attained when the following standardized criteria were met: (1) a respiratory exchange ratio of greater than or equal to 1.10; (2) failure of heart rate to increase with increases in workload; (3) post-exercise blood lactate ≥8.0 mmol•L^−1^; (4) clear signs of exhaustion (facial flushing, unsteady gait) and (5) refusal to carry on despite strong verbal encouragement (32). Maximal oxygen consumption (VO_2_ max) was determined by both aquatic and land incremental tests with stationary running to test to volitional exhaustion. HR, percentage of VO_2_ max (%VO_2_ max), percentage of HR max (% HR max), measured as the highest value obtained were recorded between the two environments. Blood lactate was measured via capillary blood sampling from the fingertips with a portable analyzer (Lactate Plus, Nova Biomedical, Waltham, Massachusetts). Data collected from the incremental test were used to determine the intensity required for the exercise interventions for each participant.

Experimental data were then collected during the AHIIT or resistive AHIIT exercises, which consisted of 5 work-rest cycles lasting a total of 10 min. REE, RER, and VO_2_ peak were continuously recorded using the PNOE breath-by-breath analyzer. Maximal and average HR were recorded using the Polar OH sensor while connected to PNOE, and RPE was recorded 15 s before transitioning to the next work/rest stage. REE referred to the energy expenditure prior to exercise with an RPE of 6 while immersed in water, as well as 2 min after the 10-minute intervention. TEE represented the cumulative energy expenditure during the 10 min of HIIT exercise, recorded and calculated using PNOE software. VO_2_ peak and mean RER were also continuously measured by PNOE. HR max and HR mean were measured by the Polar OH sensor and then recorded and analyzed using PNOE software.

### Statistical analysis

Descriptive statistics were first computed for the demographic data and a series of Shapiro-Wilk's tests were conducted to evaluate the normality of the data distributions. Continuous data measures were then summarized with means and SDs. Analyses were performed using the Statistical Package for Social Sciences for Windows version 22.0 (SPSS, Inc., Chicago, IL.). Statistical significance was delimited at *P* < 0.05. Descriptive statistics were computed and normal distributions of all variables were assessed with the Shapiro-Wilk test and the homoscedasticity was assessed with the Levene test. Paired t test was used to compare paired data. All continuous variables are presented as means and standard deviation. Mean differences among groups (AHIIT and resistive AHIIT) for each primary and secondary outcome were tested by mixed model repeated measures ANOVA. Mixed effects models were applied to analyze the effects of group (AHIIT vs. resistive AHIIT), time (pre and post interventions), and the interaction between group and time on both outcomes. Turkey post-hoc analysis was used to analyze within-group and between-group comparisons.

For the regression analysis, simple linear regression was used to predict RER from RPE in our subjects. Statistical analysis showed that RPE was a significant predictor of RER. Our assumptions for regression analysis was we assumed each of the distribution is normal and their standard deviation are equal. A visual histogram inspection was used to assess normality while plotting residuals against time can help visualize independence. The regression line is the line of best fit presented in a scattered plot. A scattered plot indicates homoscedasticity, and with the boxplots we could identify the outliers in the residuals. We discarded outliers (RER score beyond three standard deviations from the mean) prior our regression analysis.

## Results

### Subject characteristics

There was a total of 20 subjects with 11 males and 9 females. The mean age of the participants was 24.27 ± 6.59 years for male and 25.44 ± 4.22 years for females. Their average height was 174.27 ± 6.40 cm for male and 161.44 ± 4.50 cm for female. Male and female average weight was 66.64 ± 9.11 kg and 57.28 ± 9.02 kg respectively. All the participants completed 4 stages of incremental tests and training protocol.

### Results of primary outcomes

**Figure 4:**
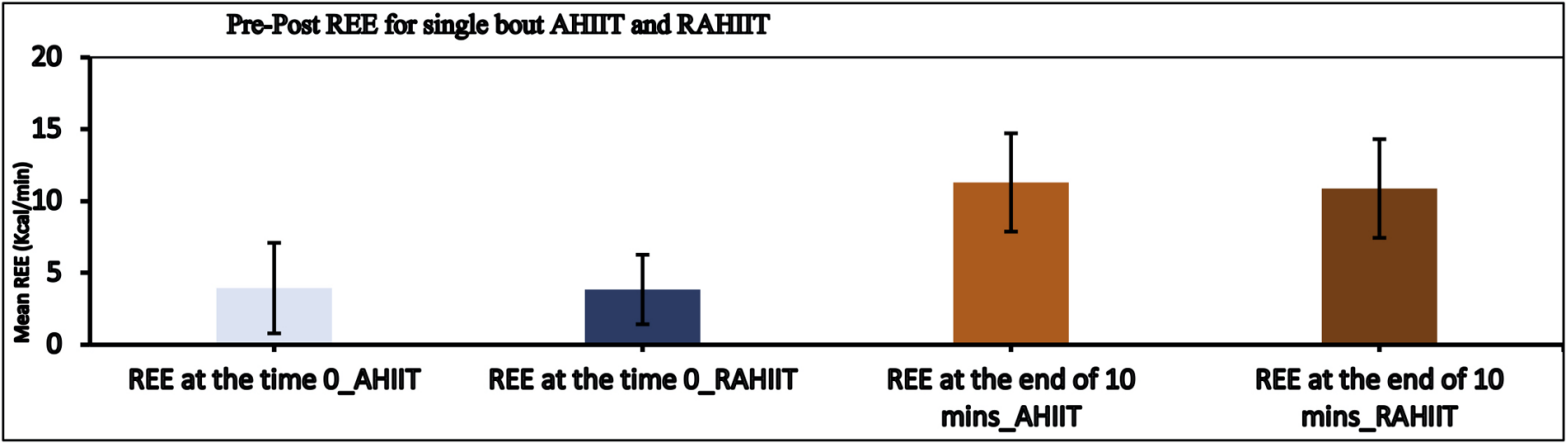
Pre and post resting energy expenditure (REE) difference in AHIIT and Resistive AHIIT (mean ± SD).

**Figure 5:**
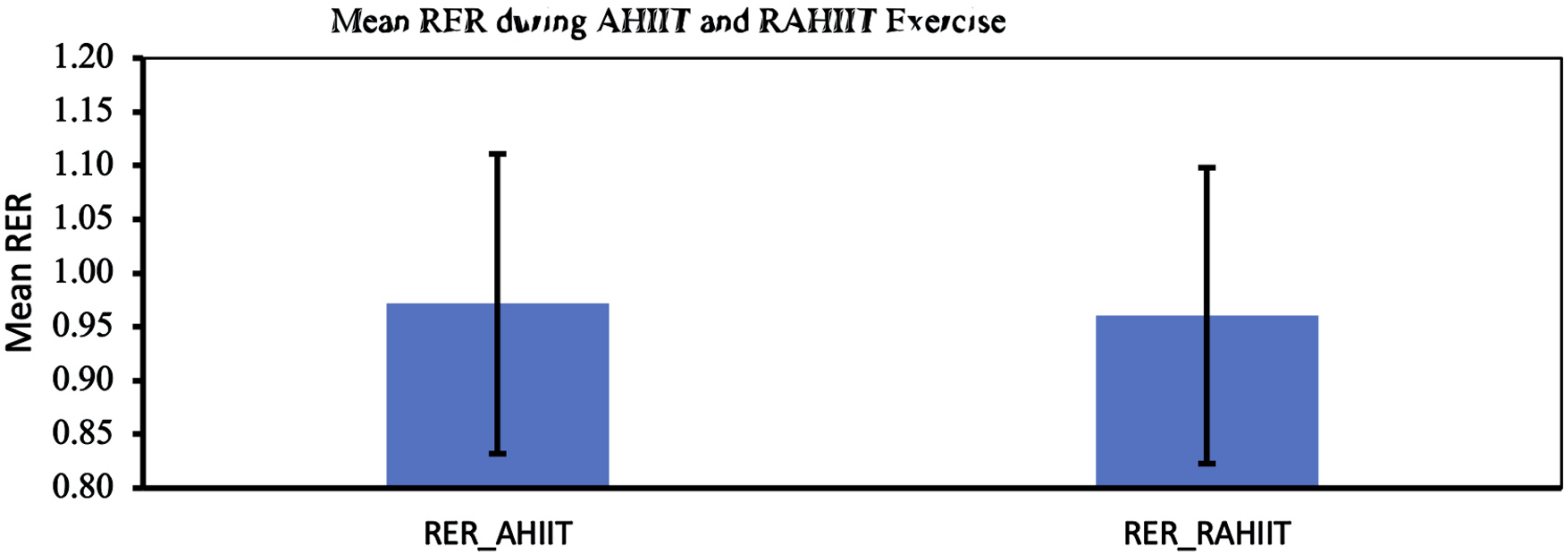
Mean respiratory exchange ratio (RER) during AHIIT and Resistive AHIIT exercise (mean ± SD).

REE within group pre-post were found to be significantly different (*p* < 0.001 and *p* < 0.001 for AHIIT and resistive AHIIT groups respectively). However, change in REE between groups do not have significant difference (*p* = 0.875 and *p* = 0.332 for AHIIT and resistive AHIIT groups respectively) (Figure 4). Mean RER between AHIIT and resistive AHIIT exercise were not found to have significant between group difference [F (1,38) = =0.615] (Figure 5). Group-by-time interactions revealed an insignificant difference in REE and RER [F (1,38) = 0.17, *P* = 0.76, ŋ^2^ = 0.01] and RER [F (1,38) = 0.02, *P* = 0.88, ŋ^2^ = 0.01] between AHIIT and resistive AHIIT (Table 1).

**Table 1:** Primary outcomes in AHIIT and resistive AHIIT (mean ± SD).

Parameters	AHIIT (*n* = 20)		Resistive AHIIT (*n* = 20)		Time effect			Group ^a^time effect		
	Pre-intervention	Post-intervention	Pre-intervention	Post-intervention	F	*P* value	ES	F	*P* value	ES
REE (Kcal/min)	3.95 ± 3.15	11.30 ± 3.42^a^	3.85 ± 2.42	10.88 ± 3.43^a^	14.99	<0.01	0.29	0.17	0.76	0.01
RER	1.1 ± 0.15	0.97 ± 0.14^a^	1.1 ± 0.16	0.97 ± 0.13^a^	7.04	<0.05	0.17	0.02	0.88	0.01

REE, resting energy expenditure; RER, respiratory exchange ratio; ES, effect size.

^a^ Time effect difference upon pairwise comparison (*p* < 0.05).

### Results of secondary outcomes

**Figure 6:**
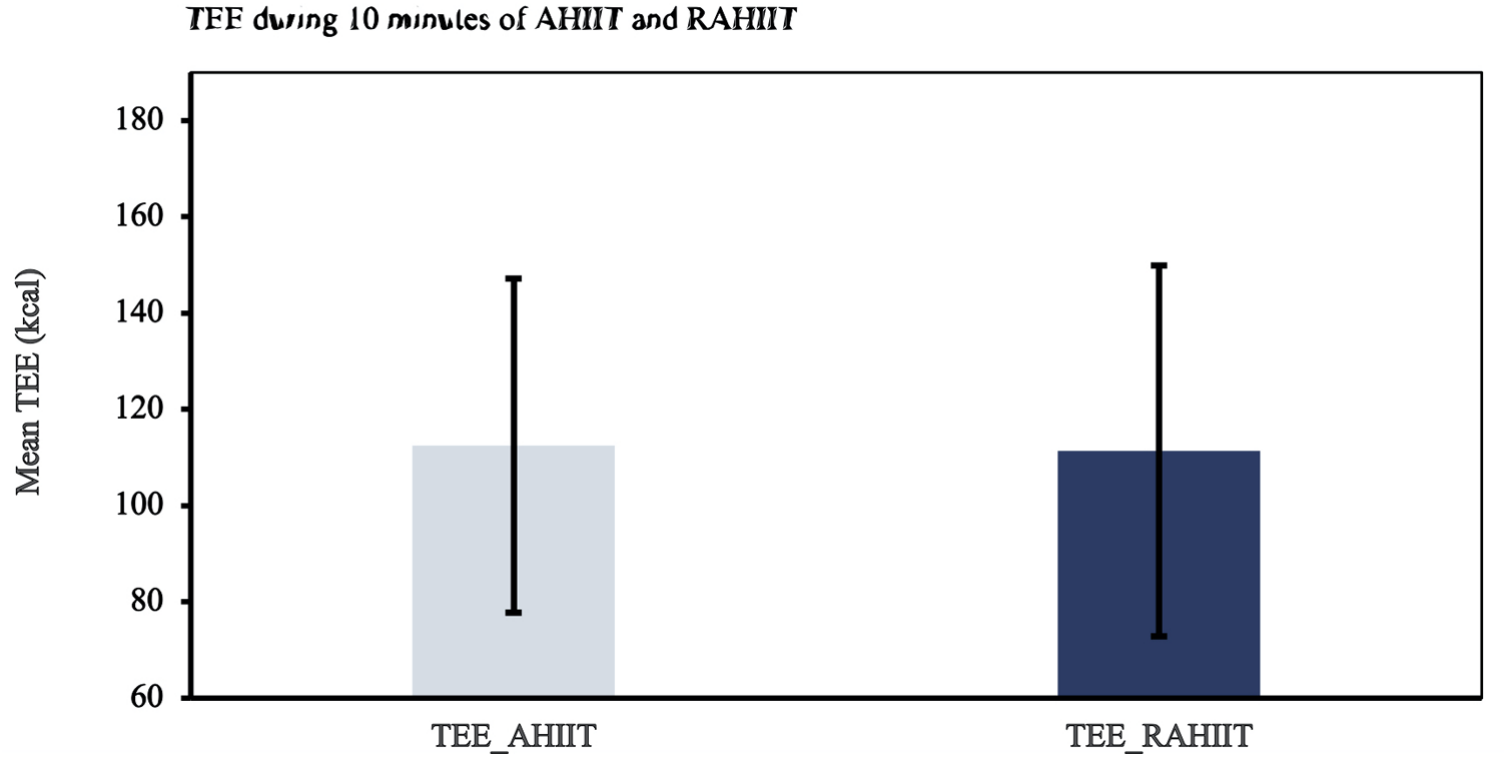
Total energy expenditure (TEE) during ten minutes of AHIIT and Resistive AHIIT exercise (mean ± SD).

**Figure 7:**
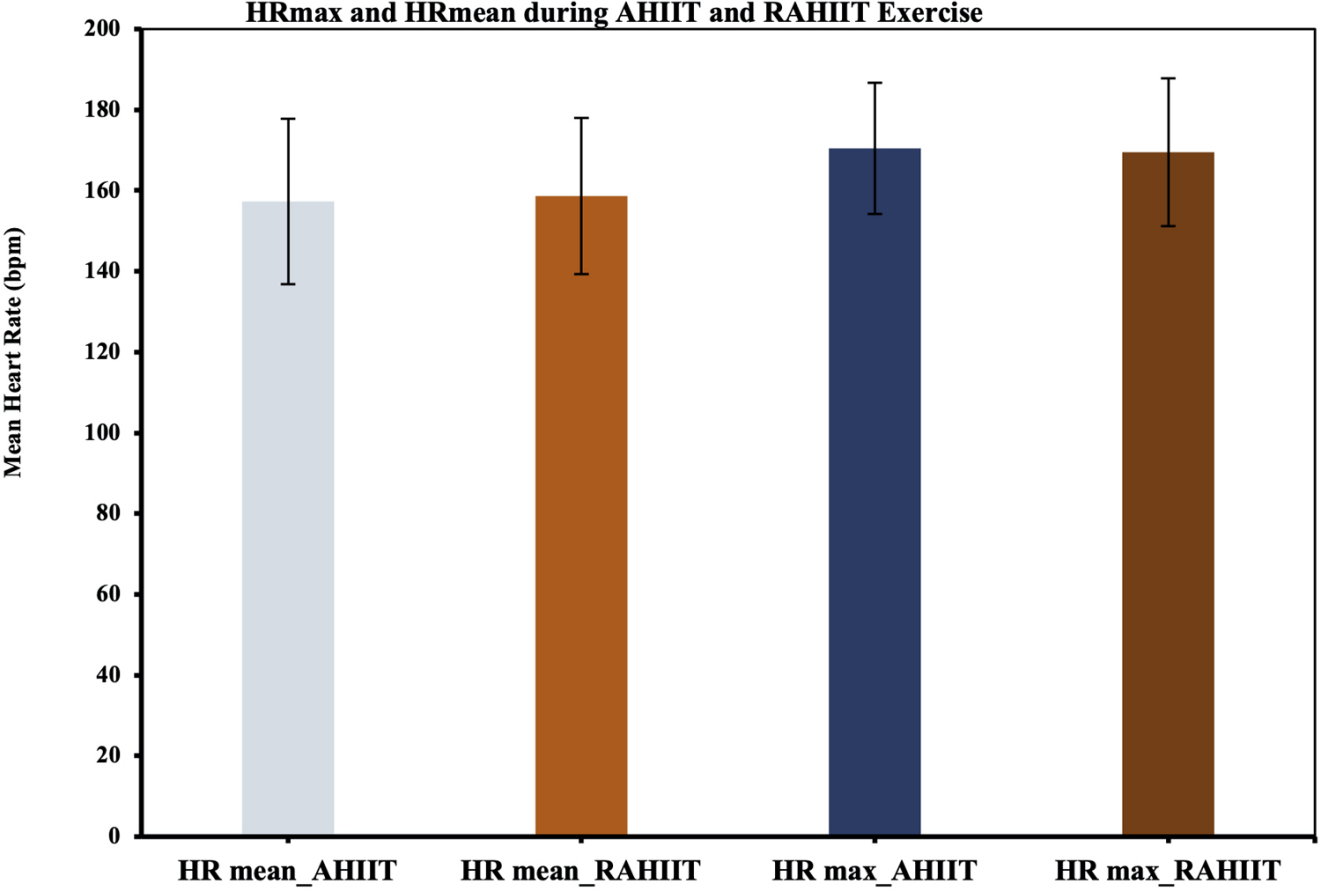
Maximal heart rate (HR max) and HR mean during AHIIT and Resistive AHIIT exercise (mean ± SD).

TEE of 10 min of AHIIT and resistive AHIIT exercise were not found to be significantly different (*p* = 0.782) (Figure 6). HR max and HR mean during 10 min of AHIIT and resistive AHIIT exercise were not found to have significant between group differences (*p* = 0.578 and *p* = 0.615 respectively) (Figure 7). None of the secondary outcomes exhibited a significant difference in the group-by-time interactions or between groups. TEE [F (1,38) = 0.81, *P* = 0.59, ŋ^2^ = 0.001], HR max [F (1,38) = 0.01, *P* = 0.50, ŋ^2^ = 0.012] and HR mean [F (1,38) = 0.08, *P* = 0.48, ŋ^2^ = 0.52]. However, there was a significant interaction shown in TEE when gender was added as covariates (ES: 0.38, *p* < 0.01).

**Figure 8:**
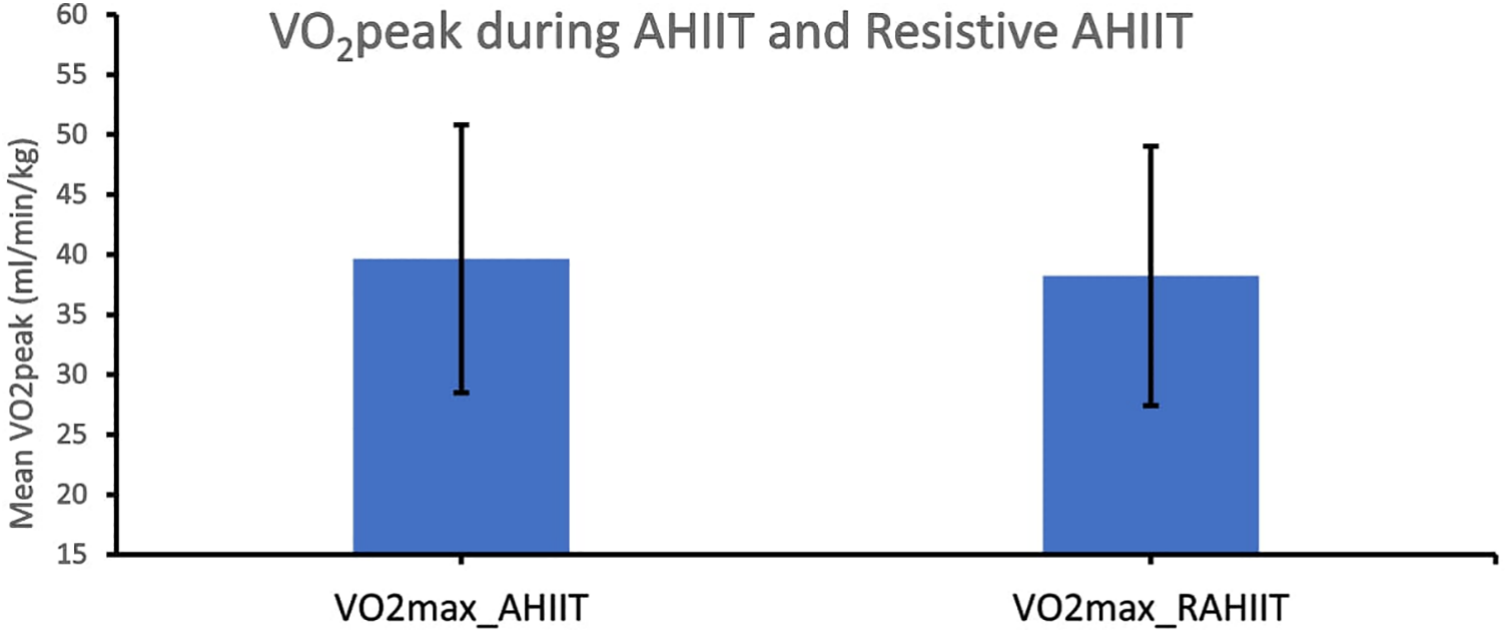
Peak aerobic power (VO_2_ peak) during AHIIT and Resistive AHIIT exercise (mean ± SD).

VO_2_ peak during 10 min of AHIIT and resistive AHIIT exercise were not found to have significantly difference between groups (*p* = 0.449) (Figure 8). RPE before and after exercise were significantly different for AHIIT and resistive AHIIT exercises (*p* < 0.01 and *p* < 0.01 respectively). AHIIT and resistive AHIIT exercise were not found to have significant between group difference (*p* = 0.615). Group-by-time interactions revealed an insignificant difference in VO_2_ peak [F (1,38) = 0.00, *P* = 0.81, ŋ^2^ = 0.06] between AHIIT and resistive AHIIT (Table 2). However, there was a significant interaction shown in VO_2_ peak when gender was added as covariates (ES: 0.37, *p* < 0.01).

**Table 2:** Secondary outcomes in AHIIT and resistive AHIIT (mean ± SD).

Parameters	AHIIT (*n* = 20)		Resistive AHIIT (*n* = 20)		Time effect			Group ^a^time effect		
	Pre-intervention	Post-intervention	Pre-intervention	Post-intervention	F	*P* value	ES	F	*P* value	ES
TEE (Kcal)	113.26 ± 57.84	101.20 ± 37.66	123.84 ± 60.32	103.72 ± 35.74	0.78	0.23	0.04	0.81	0.59	0.001
HR max (bpm)	178.20 ± 14.94	170.45 ± 16.25	173.20 ± 15.52	169.75 ± 18.04	2.73	0.15	0.07	0.01	0.50	0.012
HR mean (bpm)	146.3 ± 16.43	160.25 ± 18.19	143.70 ± 17.39	161.70 ± 16.53	0.52	0.20	0.05	0.08	0.48	0.52
VO_2_ peak (ml/min/kg)	42.61 ± 9.98	39.65 ± 11.16	43.21 ± 11.08	39.61 ± 9.57	0.27	0.63	0.24	0.00	0.81	0.06
RPE	6.00 ± 0.00	13.70 ± 1.72^a^	6.00 ± 0.00	14.75 ± 2.45^a^	35.76	<0.01	0.49	2.63	0.11	0.07

TEE, total energy expenditure, HR max, maximal heart rate, HR mean, mean heart rate, VO_2_ peak, peak oxygen consumption, RPE, rate of perceived exertion, ES, effect size.

^a^ Time effect difference upon pairwise comparison (*p* < 0.05).

**Figure 9:**
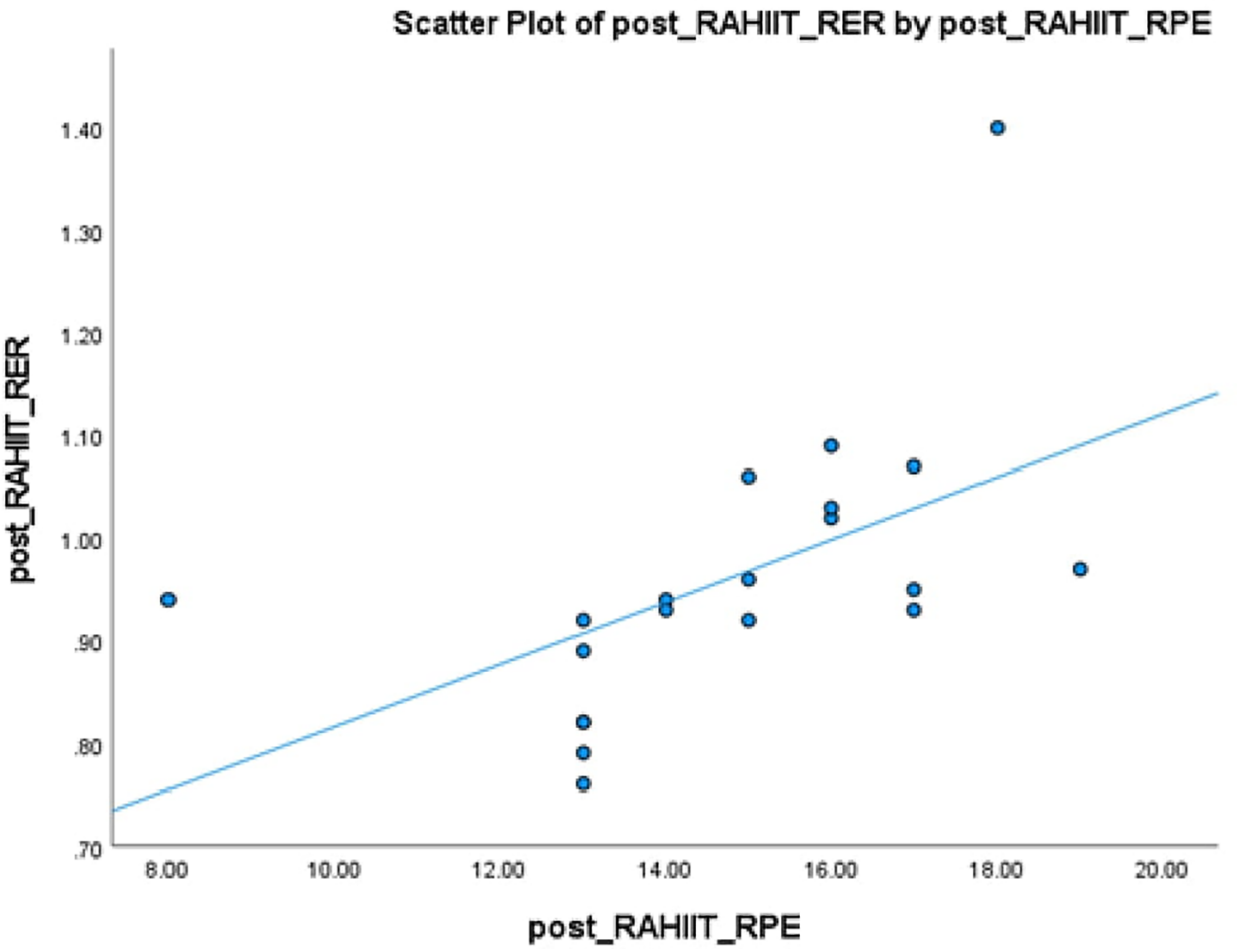
Correlation between the rate of perceived exertion (RPE) and respiratory exchange ratio (RER), (mean ± SD), r = 0.543.

Simple linear regression was used to predict RER from RPE. Simple linear regression model was used to show the best adjustment in all analysis, with significant relationship (*p* = 0.013) observed between subjective and the metabolic variables between RPE and RER (Figure 9). The R (0.543) was shown to be statistically significant, the RPE is a significant predictor of RER. And r values were classified according to the recommendations from Safrit-and Wood (33), i.e., 0–0.19 as no correlation, 0.2–0.39 as low correlation, 0.4–0.59 as moderate correlation, 0.6–0.79 as moderately high correlation, and 0.8–1.0 as high correlational analyses.

## Discussion

To the best of the author's knowledge, this study represents the first attempt to investigate the impact of AHIIT and resistive AHIIT on metabolic and cardiorespiratory responses in healthy adults. The objectives of this study were to determine whether an acute session of resistive AHIIT would produce comparable or increased metabolic and cardiorespiratory responses compared to AHIIT at the same training intensity. The key findings of this study are summarized as follows: 1. Both AHIIT and resistive AHIIT were associated with higher resting energy expenditure (REE) following a single session, but no significant differences were observed between the two groups. 2. Significant differences were observed between the two groups in terms of rating of perceived exertion (RPE), with resistive AHIIT showing a higher RPE. Additionally, there was a moderate correlation between RPE and respiratory exchange ratio (RER). 3. Both within-group and between-group analyses revealed significant pre-post differences for various outcomes. 4.Regarding the cardiometabolic outcomes of HR max, HR mean, VO_2_ peak, and TEE, no significant differences were observed between the two groups.

### Resting energy expenditure

The results of this study support our initial hypothesis that a single session of resistive AHIIT would have a comparable effect on basal metabolism as AHIIT. Both AHIIT and resistive AHIIT significantly increased post-training basal metabolism, indicating that performing high-intensity interval training in an aquatic environment enhances basal metabolic rate in general. In contrast, studies conducted in a land-based environment by Hazell et al. (34) and Skelly et al. (35) demonstrated that a single session of LHIIT significantly increased resting energy expenditure (REE) after 1 h and 24 h of exercise (34, 35). When comparing LHIIT and AHIIT, Kwok et al. (16) found no significant difference in terms of energy expenditure (17). This suggests that high-intensity interval training improves basal metabolic rate in general, regardless of the training environment. Therefore, the effect on basal metabolism should not be the sole consideration when deciding whether to perform high-intensity interval training on land or in water. However, AHIIT offers advantages such as lower heart rate, lower perceived exertion during exercise, and faster recovery rates compared to LHIIT. Factors such as cardiometabolic responses, perceived exertion, recovery rate, and the properties of water, such as buoyancy and hydrostatic pressure, should be primary considerations when choosing AHIIT over LHIIT (17, 36).

Although both AHIIT and resistive AHIIT resulted in a significant increase in REE, this study did not find evidence to suggest that resistive AHIIT would elicit a greater increase in post-training basal metabolism compared to AHIIT. This finding is consistent with the secondary outcomes, as no significant differences were observed between the AHIIT and resistive AHIIT groups in terms of HR max, HR mean, and VO_2_ peak. Potential reasons for this lack of difference can be explained by factors highlighted by Paoli et al. (19): the specific exercise selected (in this case, stationary run) directly influences the trained muscle groups and consequently the resulting REE. Additionally, the active recovery periods between sets, as well as the number of repetitions and sets, are crucial factors that determine whether a statistically significant difference in REE or other secondary metabolic outcomes can be observed (19). Therefore, it is suggested that besides exercise intensity, other training details and factors, such as the specific muscle groups targeted and the overall exercise setup, should be taken into account to achieve a higher post-training REE in a single session of AHIIT.

### Metabolic outcomes

Regarding another primary metabolic outcome, respiratory exchange ratio (RER), our study found no significant difference between the AHIIT and resistive AHIIT groups. This suggests that adding a resistive component to our originally designed AHIIT protocol did not result in a statistically significant difference in RER compared to non-resistive AHIIT. When considering other cardiometabolic outcomes such HR max, HR mean, VO_2_ peak, and TEE, the addition of resistive components did not lead to significant changes in these outcomes. This led the researchers to question whether non-resistive HIIT, which solely relies on water as a resistance medium, is sufficient to challenge RER as a metabolic marker in healthy adults.

Due to technical limitations with the PNOE analyzer, our study was only able to measure the change in RER rather than retrieving pre-post RER values for both the AHIIT and resistive AHIIT groups. Our results showed no significant difference in RER between the AHIIT and resistive AHIIT groups (*p* = 0.981). Additionally, a study by Tang et al. (37) demonstrated the pre-post RER difference in an AHIIT group compared to moderate intensity continuous training on land (37). In that study, thirty-one inactive adults were randomly assigned to either AHIIT or moderate intensity continuous training on land, and various parameters including central hemodynamics, endothelial function, and aerobic fitness were measured over a 6-week period. The results showed an effect size of −0.222 for the influence of AHIIT on RER, while the effect size for moderate intensity continuous training on land was 0.00. This indicates that neither training protocol, whether in a land or aquatic medium, produced a statistically significant effect on RER.

Despite the lack of evidence establishing a positive relationship between AHIIT and RER, AHIIT is still considered beneficial for other cardiometabolic outcomes such as HRmax, VO_2_ max, and energy expenditure when compared to continuous aerobic exercise protocols on land (6).

### Self-perceived exertion

Our study indicated that adding a resistive component to the AHIIT protocol resulted in a higher perceived exertion level, as reflected by statistically significant differences in rating of perceived exertion (RPE). However, the addition of the resistive component to aquatic HIIT did not lead to statistically significant differences in the studied cardiometabolic outcomes. In terms of RPE, there were significant differences within both the AHIIT and resistive AHIIT groups (*p* < 0.01), as well as between-group differences (*p* = 0.007). Extra drag force added by additional resistance becomes significant on load enforcement therefore even a small increase in resistance leads to a considerable rise in rate of perceived exertion (Hilman et al). A moderate correlation was found between RPE and RER in the resistive AHIIT group (r = 0.543). This result aligns with other studies that highlight RPE as an indicator of exercise intensity (13). Additionally, the RPE is a significant predictor of RER and accounted for 9.3% of the variance in RER because of maximal tests require an individual to exercise to the point of volitional fatigue or until a clinical indication to stop. Criteria have been used to confirm the maximal effort which included RPE at peak exercise >7 on the 6–20 scale or >7 on the 0–10 scale or a peak of RER ≥ 1.10.

Regarding other cardiometabolic outcomes such as REE, RER, VO_2_ peak, HR max, HR mean, and TEE, our results showed no statistically significant differences between the two groups. Since both AHIIT and resistive AHIIT demonstrated comparable effects on resting energy expenditure and cardiometabolic responses, but participants perceived a lower exertion level in AHIIT compared to resisted AHIIT at the same exercise intensity, AHIIT can be preferred as a training program to enhance participants’ exercise compliance and overall health. This finding also suggests that instead of progressing subjects with a resistive component in a water-based environment, the focus could be on monitoring subjects’ RPE and maintaining appropriate heart rates during AHIIT (17). For the effect of sex as covariate that influenced VO_2_ peak. This is primarily due to male have a higher ventricular ejection volume, hemoglobin concentration, muscle mass and lower body fat. And since as muscle is the greatest consumer of oxygen during exercise, greater muscle mass in men is responsible in part for their greater absolute VO_2_ peak compared to women (38).

Judging by the standardized regression coefficients, RPE is an important predictor of RER. Those with higher RPE tend to have greater RER, but no causal relationship can be identified due to cross sectional design.

### Beneficial effects of resistive AHIIT as reflected in other metabolic outcomes

Resistive training did not lead to statistically significant changes in resting energy expenditure (REE) and respiratory exchange ratio (RER) when compared to non-resistive HIIT. However, the researchers hypothesized that the beneficial effects of resistive training might be observed in other metabolic outcomes such as muscle fiber capillarization, muscle morphology, and succinate dehydrogenase (SDH) activity (39).

In a randomized controlled trial conducted by Leuchtmann et al. (39), twenty older recreationally active men were recruited and assigned to either 12 weeks of habitual observation followed by 12 weeks of resistance training (RT), or 12 weeks of high-intensity interval training (HIIT) followed by 12 weeks of RT (39). The results showed that both groups were equally effective in improving capillarization and oxidative enzyme activity, as assessed through biopsies of the vastus lateralis muscle. Furthermore, the RT group was able to sustain the metabolic parameters induced by the HIIT intervention. This suggests that future research could focus on examining whether there are significant differences in the aforementioned metabolic outcomes between AHIIT and resistive AHIIT, rather than relying solely on RER and REE as metabolic indicators.

### Strengths and limitations

This study has several strengths. The novelty of an incremental test performed prior to the AHIIT and resistive AHIIT intervention allowed a correct, optimal and matched intensity of REE, RER, HR mean,%HR max,%VO_2_ max, RPE and monitoring in both groups for comparison. The aquatic incremental tests were conducted in both non-resistive and resistive groups, which were considered the most accurate methodology for exercise intervention as these allowed the standardization of water as the medium as well as previously aforementioned properties (i.e., buoyancy, hydrostatic pressure, drag forces) of water compared to conducting the incremental tests on land (5). By using the HR matched cadence for determining the equivalent intensity for 90% and 70% HR max, this could individualize the training intensity for healthy adults and to serve as the baseline measure for later cardiometabolic evaluation.

Despite these strengths, major limitations of the present study included small sample size and hence caution should be taken when generalizing to the older population. Moreover, the small sample size of 20 subjects might hinder the generalization to the clinical populations. Regarding the design of this study which was a cross-sectional study for examining the instantaneous effect of AHIIT and resistive AHIIT only, the long-term effect of these interventions on cardiometabolic outcomes were yet to be proven. Nevertheless, our results provide practical guidelines in applying matched intensity of aquatic incremental tests followed by the HIIT corresponding interventions. From a practical point of view, the HR matched cadence could allow healthy subjects to achieve training intensity equivalent to 90% and 70% of HRmax in water medium. The distinctive characteristics of water like buoyancy and hemodynamic properties enabled subjects to enjoy physiological advantages of AHIIT. A randomized control study could be suggested studying the long-term effect of AHIIT on cardiometabolic outcomes which might yield different results from the single bout session.

## Conclusion

In summary, our findings indicate that both AHIIT and resistive AHIIT result in significant differences in RPE, while showing no significant differences in other metabolic and cardiorespiratory responses such as HR max, HR mean, VO_2_ peak, and TEE. The moderate correlation between RPE and RER in resistive AHIIT suggests that RPE can serve as an indicator for prescribing exercise intensity effectively. The addition of a resistive component to AHIIT can yield comparable results to using water's drag force as the sole medium for resistance. AHIIT still offers cardiometabolic benefits when compared to resistive AHIIT. Therefore, future research should focus on conducting randomized controlled trials to examine the long-term effects of comparing AHIIT with resistive AHIIT.

## Data Availability

The raw data supporting the conclusions of this article will be made available by the authors, without undue reservation.
